# Focal nodular hyperplasia on an accessory liver lobe: A case report and literature review

**DOI:** 10.1097/MD.0000000000021357

**Published:** 2020-07-24

**Authors:** Na Su, Cheng Chen, Qing Dai, Liang Wang, Meng Yang, Yu Xia, Yuxin Jiang, Ke Lv

**Affiliations:** Department of Ultrasound, Peking Union Medical College Hospital, Chinese Academy of Medical Sciences and Peking Union Medical College, Beijing, China.

**Keywords:** accessory liver lobe, case report, focal nodular hyperplasia, integrated radiologic approach

## Abstract

**Introduction:**

Accessory liver lobe (ALL), an autonomous island of normal liver parenchyma, is a rare congenital anomaly that is difficult for preoperative diagnosis and often identified incidentally. It can also be accompanied with benign or malignant diseases, which is extremely rare. There are only 3 cases of focal nodular hyperplasia (FNH) detected on ALL reported by previous literature.

**Patient concerns:**

A 33-year-old woman was incidentally diagnosed with a mass in left upper quadrant abdomen by a routine ultrasound examination. Doppler ultrasound revealed that the mass was attached to left liver lobe with a vascular pedicle. A spoke-wheel artery with diffuse enhancement during hepatic arterial phase was visualized on contrast-enhanced ultrasound, and the mass was continuously hyper-enhanced with a hypo-enhanced intralesional scar during the portal and delayed phase. And contrast-enhanced computed tomography showed a similar enhancement mode of the mass.

**Diagnosis:**

The mass was resected and postoperative histopathologic result of the lesion revealed a nodular hyperplastic parenchyma with a central fibrous scar, without tumor cells. And a final diagnosis of FNH on ALL was determined accordingly.

**Interventions:**

Mass resection was conducted according to patient's demand.

**Outcome:**

After general postoperative administration, the patient was discharged. Then, she had been undergoing regular serological tests and imaging examinations in our hospital for 24 months.

**Conclusion:**

The finding of a mass connecting with liver by a stalk should alert the clinician of the possibility of ALL, as well as benign or malignancies on an ALL. This is the first case of FNH on ALL preoperatively confirmed by contrast-enhanced ultrasound. We suggest that an integrated radiologic approach is crucial to evaluate an incidentally detected, asymptomatic abdominal focal mass.

## 1 Introduction

Accessory live lobe (ALL) is a rare congenital liver anomaly, whose incidence is 0.09% based on data described in studies of laparoscopy and autopsy.^[1]^ Preoperative diagnosis of ALL is highly challenging, as most of them are asymptomatic unless a pedicle torsion, compressive effect, or neoplastic change occurs.^[2–4]^ ALL may have pathologic features similar to anatomically normal liver, thus leading to the incidence of benign or malignant diseases that appear in liver tissues, which is an extremely rare condition. Benign diseases on ALL, including hemangioma, focal nodular hyperplasia (FNH) and adenoma, have lower incidence than that of malignancies. Only 3 FNH on ALL cases have been reported in English literature.^[3–5]^ Herein, we presented case of an asymptomatic mass that was verified as FNH on an ALL finally in this study, and we paid special attention on its imaging presentations.

## 2 Case presentation

**Figure 1:**
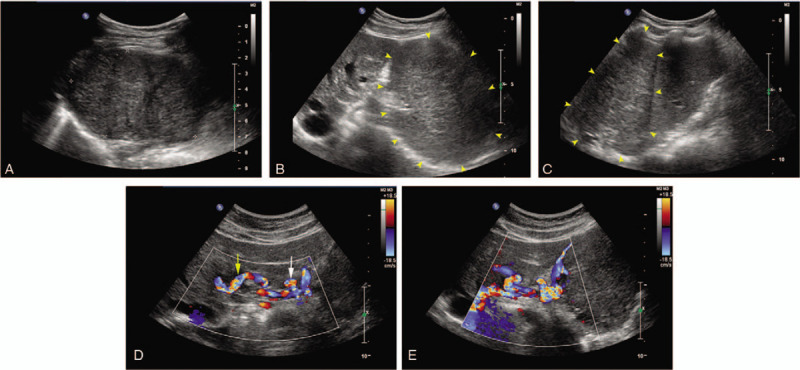
A. Gray scale ultrasound: a well-defined hypoechoic mass was found (calipers). B. Gray scale ultrasound: the mass (yellow arrow heads) was lateral to the left liver lobe. C. Gray scale ultrasound: the mass (yellow arrow heads) was close to the spleen, relative movement was present during respiration. D.E. The mass was highly vascularized by Doppler, a vascular pedicle was found between the mass (white arrow) and liver (yellow arrow).

A 33-year-old woman was incidentally diagnosed with a mass in left upper quadrant abdomen by a routine ultrasound scanning, with no specific personal and family history. For physical examination, there was no palpable mass, tenderness, or rebound tenderness. Laboratory findings including HBV and HCV tests, amylase, liver function tests autoantibodies and tumor markers (serum α-fetoprotein, CA 199 and CA 125) were normal. Doppler ultrasound demonstrated an enlarged and distorted artery in her left liver lobe. By tracing the vessel, a well-defined hypoechoic mass was found outside the left liver lobe, 83 mm × 59 mm × 56 mm in diameter (Figs. 1A and 1B). The mass was close to the spleen, relative movement was present during respiration (Fig. 1C), and the mass was highly vascularized by Doppler (Figs. 1D and 1E).

**Figure 2:**
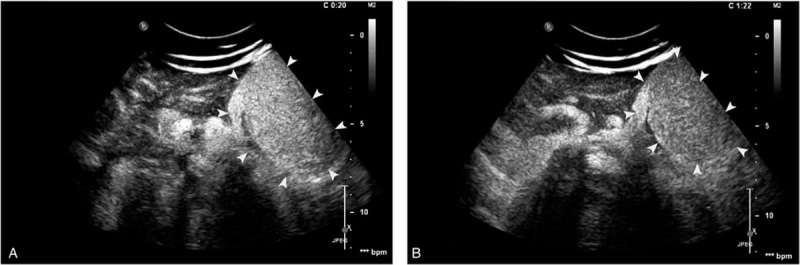
A. contrast-enhanced ultrasound: a remarkable quick enhancement of the mass (white arrow heads) compared to the liver in the arterial phase. B. contrast-enhanced ultrasound: the enhancement of the mass (white arrow heads) was almost isoechoic to the liver parenchyma during the portal and late phase.

We performed Contrast-enhanced ultrasound (CEUS) using an intravenous dose of 2.4 mL SonoVue (Bracco Inc., Milan, Italy) for further investigation. Regarding the normal liver tissue as control, the mass had a remarkable quick wash-in during hepatic arterial phase (HAP), with a centrifugal enhancement pattern (Fig. 2A). Continuous hyper-enhancement with a hypo-enhanced intralesional scar was seen in the portal and delayed phase (Fig. 2B). A second dose of 1.2 mL of SonoVue was then administered, the blood supply of the mass was found arising from a vascular pedicle, which originated from distorted artery inside the left liver. Furthermore, the venous flow of the mass was found to drain into enlarged left hepatic vein through the connected stalk.

**Figure 3:**
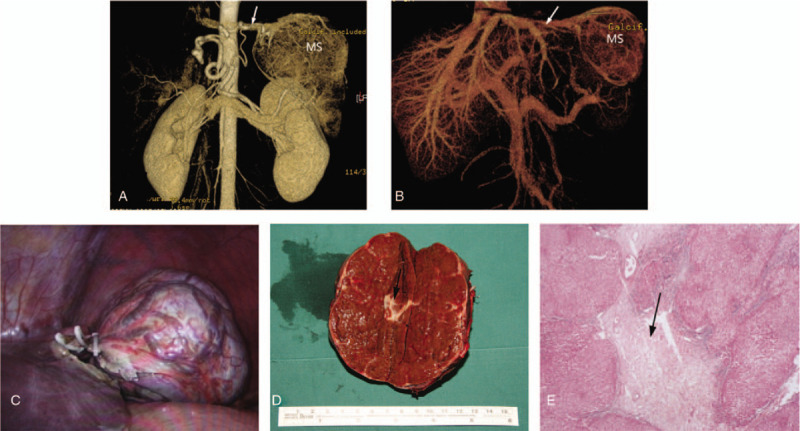
A. volume rendering technique image: the mass (MS) was perfused by a single distorted artery (white arrow) directly originating from the aorta. B. volume rendering technique image: the blood (white arrow) from the mass (MS) drained into the enlarged left hepatic vein. C. D. Intraoperative image: abdominal mass localized in the under surface of the liver and attached to the lateral segment of the left hepatic lobe through a pedicle, which was located in the left liver triangular ligament, with a fibrous capsule and multiple blood vessels on its surface, with a central fibrous scar (black arrow). E. Photomicrograph: objective light microscopic image of hematoxylin-eosin-stained specimen slide from this patient in at low-power magnification reveals a nodular hyperplastic parenchyma with a central fibrous scar (black arrow), containing a proliferation of small bile ducts and irregular tortuous arteries with thickened walls, veins, and capillaries.

Contrast-enhanced computed tomography (CECT) confirmed the presence of a large and well-defined abdominal mass between spleen and diaphragm, attached to the left liver lobe with a stalk. In the arterial phase, the mass was avidly and homogeneously enhanced with a central irregular hypodense area. Moreover, multi-slice spiral computed tomographic angiography (MSCTA) and volume rendering technique images suggested that the blood supply of the mass originated from a single distorted artery directly from the aorta (Fig. 3A), with venous flow draining into left hepatic vein (Fig. 3B).

Initially diagnosing the mass as FNH in an ALL according to the imaging manifestation, we performed laparotomy subsequently which revealed that the mass was located in the left liver triangular ligament, connecting to the lateral segment of the left hepatic lobe through a pedicle. The mass was covered with a fibrous capsule and multiple blood vessels. A central fibrous scar was also found by laparotomy (Figs. 3C and 3D). Postoperative histopathology of the mass demonstrated a nodular hyperplastic parenchyma with a central fibrous scar, containing proliferation of small bile ducts and irregular tortuous arteries with thickened walls, veins and capillaries, and no tumor cell was detected by histology (Fig. 3E). Therefore, the histopathological result confirmed the diagnosis of classical FNH on an ALL.

After general postoperative administration, the patient was discharged and received regular serological tests and imaging examinations in our hospital for 24 months. The patient has provided informed consent for publication of the case.

## 3 Discussion

Accessory liver tissue is an autonomous island of normal liver parenchyma. They may be located at various sites, most frequently on the inside of the gallbladder, and may also be involved with ligaments around liver, spleen, portal vein, umbilicus, adrenal grand, pancreas, esophagus, diaphragm, and thoracic cavity.^[2,5]^

There are 2 types of accessory liver lesions. The first is an accessory lobe connected to the liver, named as accessory live lobe (ALL), which was seen in 0.09% of patients. And the second is the ectopic liver completely separated from the liver, which was seen in 0.47% of patients.^[1]^ However, it is difficult to make a clear distinction between the 2 types in many cases, while the precise incidence is unknown.

ALL is subject to the same benign or malignant diseases as the original liver tissues,^[4]^ which may be caused by abnormalities of vascular supply and biliary drainage.^[3]^ It is reported that ALL associated with benign lesions are rarer than with malignancies, benign diseases that have been reported including hemangioma, FNH, and adenoma.^[4]^

In this case, an abdominal mass was found between left liver lobe, spleen, and diaphragm during routine examination. Grey scale and Doppler ultrasound revealed that the mass was attached to left liver lobe by a vascular pedicle, with its characteristics unknown. Furthermore, CEUS showed a spoke-wheel artery with diffuse enhancement during HAP, and the mass was continuously hyper-enhanced with a hypo-enhanced intralesional scar in the portal and delayed phase. These radiologic findings highly suggested the diagnosis of FNH. As the mass was outside but connected to liver by a stalk, it was considered as FNH on an ALL, which was finally proved by pathology.

We searched the database, including PubMed, Scopus, and Excerpt Medica Database (EMBASE), to identify studies evaluating FNH on ALL. According to previous literature, only 3 cases of FNH on ALL have been reported in English literature (Table 1). These 4 patients had different signs when referring to hospital, of whom 2 had abdominal pain, 1 had an isolated increase in GGT. These lesions were found in areas between spleen and diaphragm, between kidney and liver, and in the pelvis. Clinical manifestations were depended on compression of adjacent organs, the location, and pedicle torsion of ALL.^[6,7]^

**Table 1:**
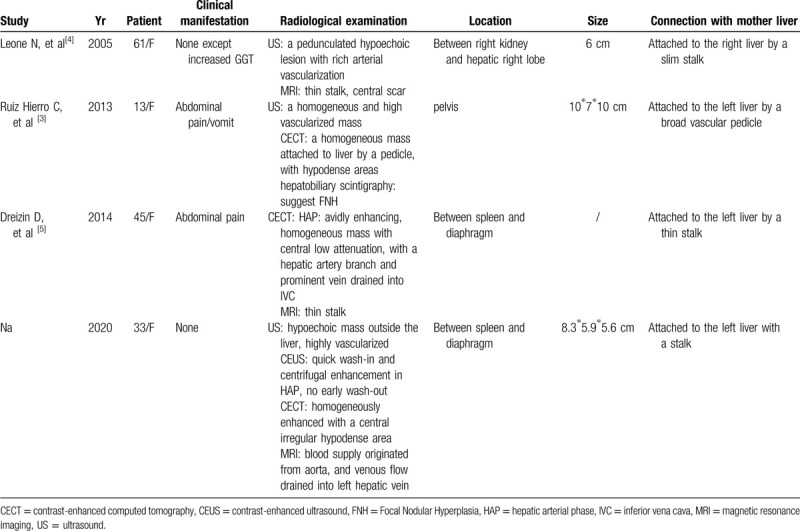
Cases of focal nodular hyperplasia on an accessory hepatic lobe.

Demonstrated by imaging results, the 3 lesions and the case in our study were all connected to liver tissue by a vascular pedicle. They had large hepatic artery branch and a prominent vein, which drained directly into the hepatic vein or inferior vena cava. Specific imaging features were visualized in CEUS and CECT imaging, such as diffuse hyper-enhancement during HAP, continuous hyper-enhancement with hypo-enhanced intralesional scar in the portal and delayed phase. Totally, all the 4 lesions can be considered as FNH on an ALL by imaging examinations before surgery.

To our knowledge, we present the first case of FNH on ALL pre-operatively which was confirmed by CEUS. CEUS showed a blood vessel arising from aorta, distorted within the liver, and then connected to the extrahepatic mass by a vascular pedicle, consistent with intra-operative observation. However, MSCTA could only indicate the vascular pedicle arose from the aorta, and the vessel distortion through the liver could not be seen, for liver tissue could not be visualized by MSCTA. CEUS is a minimally invasive method for preoperative evaluation of abdominal masses. They can show a real-time vascular map of the mass, also the relationships with organs, thus indicating the characteristics of the blood supply. Moreover, the microbubbles used as the contrast agents for CEUS possess high safety, and seldom cause hepatotoxicity and allergies. Therefore, as a non-ionizing and non-radiative imaging method, CEUS can be an alternative to CT/CECT, especially for pediatric patients, for whom radiative imaging methods are not suitable. Experienced radiologists are needed to perform CEUS, which is the main limitation of the novel method, but its wide clinical utilization can still be expected.

## 4 Conclusion

In conclusion, we supposed that FNH on an ALL can be diagnosed by integrated radiological examinations before surgery. When a mass is found in a special location of the abdominal or pelvic cavity, it is necessary to use integrated imaging approaches to evaluate the lesion. If the mass is outside but attaches to the liver with a vascular pedicle, it is highly indicated as an ALL. In the meanwhile, we should better maintain an awareness of the possibility of benign or malignancies on an ALL. Further diagnostic confidence may be achieved with the use of CEUS, which provides clear vascular map in a non-invasive way.

## Author contributions

**Data curation:** Na Su, Cheng Chen.

**Resources:** Qing Dai.

**Software:** Cheng Chen.

**Supervision:** Liang Wang, Yu Xia.

**Visualization:** Meng Yang.

**Writing – original draft:** Na Su.

**Writing – review & editing:** Cheng Chen, Yuxin Jiang.
